# Ethical conflicts experienced by community nurses: A qualitative study

**DOI:** 10.1177/09697330231200563

**Published:** 2023-10-05

**Authors:** Caroline Porr, Alice Gaudine, Joanne Smith-Young

**Affiliations:** Faculty of Nursing 7512 , Memorial University of Newfoundland

**Keywords:** Home care, qualitative research, ethics of care/care ethics, moral distress, end of life issues, community care

## Abstract

**Background:**

Despite news reports of morally distressing situations resulting from complex and demanding community-care delivery in Canada, there has been little research on the topic of ethical conflicts experienced by community-based health care professionals.

**Research aim:**

To identify ethical conflicts experienced by community nurses.

**Research design:**

Data were collected using semi-structured interviews and then relevant text was extracted and condensed using qualitative content analysis. This research was part of a larger grounded theory project examining how community nurses manage ethical conflict.

**Research context and participants:**

Community nurses, including 13 public health nurses and 11 home care nurses from two Canadian provinces, were interviewed.

**Ethical considerations:**

Study approval was granted by the Health Research Ethics Authority of Newfoundland and Labrador and by provincial health authorities.

**Findings:**

Seven ethical conflicts were identified and assigned to one of two groups. In the grouping categorized as challenges with obligations or risks, the ethical conflicts were: (1) screening for child developmental issues knowing there is a lack of timely early intervention services; (2) encountering inequities in the health care system; (3) not fulfilling principles, goals, and initiatives of primary and secondary prevention; and (4) feeling powerless to advocate for clients. The remaining ethical conflicts were categorized as challenges with process, risks, and consequences, and were: (5) jeopardizing therapeutic relationships while reporting signs of a child at risk; (6) managing confidentiality when neighbors are clients; and (7) supporting client autonomy and decision-making but uncertain of the consequences.

**Conclusions:**

Research investigation will continue to be important to raise awareness and mobilize ethics supports as health care services are steadily shifted from institutional to community settings. Moreover, with heightened potential for communicable disease outbreaks across international borders from global warming, community nurses around the world will continue to be required to address ethically-difficult care situations with competence and compassion.

## Introduction

Community nurses are community-based health care professionals who provide important and diverse services and programs in a variety of settings. While the significance and stressors of their work was highlighted around the world during the pandemic, little research has been conducted in Canada on the everyday ethical challenges facing community nurses; rather, researchers have focused on the ethical issues of acute care nurses. As community-care delivery expands in Canada, nurse leaders and educators need to be informed of the ethical conflicts that do and can arise in community nursing practice. It is only then that community nurses can be better supported and nursing students adequately equipped to fulfill complex and demanding roles.

## Background

When community nurses perceive that their course of action or decisions made by their organization may violate their practice values and moral integrity, they experience an ethical conflict.^1^ For nurses in Canada, ethical values and moral integrity are informed by the *Canadian Nurses Association Code of Ethics for Registered Nurses*.^2^ Community nursing in Canada is a specialty area. Nurses may work in one of two position streams: as a home care nurse (HCN) responsible for in-home care and support or as a public health nurse (PHN) contributing to health promotion and to disease, disability, and injury prevention. A HCN visits patients of all ages in the home setting providing medical care such as medication administration to address disease, disability, and palliation, or to monitor wound healing, and, may assist with personal grooming and mobilization. A PHN works with individuals of all ages, and families, groups, and communities, often delivering education, health screening, and immunization programs, and, coordinating population health assessment and surveillance, and emergency preparedness.

Given the broad scope of roles and responsibilities of community nurses, undoubtedly, they experience ethical conflicts. However, little has been conducted on the topic of ethical conflicts in community nursing practice and to our knowledge there are no published studies from Canada despite the fact there have been several national news stories of seemingly morally distressing situations encountered by community nurses and other community-based health care professionals. CBC News reported, for example, on community nurses having to deal: with seniors being placed in separate nursing homes;^3^ with new provincial legislation forcing families to transfer their parents who are older adults from hospital settings to long-term care facilities, not of their choosing;^4^ and with persons being denied in-home palliative care services.^5^

In other countries there has been research exploring ethical dilemmas encountered by community nurses who provide palliative care in hospice settings, for example in Italy,^6^ and who promote autonomy (e.g., in another study in The Netherlands) of clients who are older adults or chronically ill or living with disabilities.^7^ Researchers in Norway studied the everyday ethics of home care managers and discovered that several ethical challenges faced by HCNs were associated with resource allocation and fiscal constraints.^8^ Also from Norway, researchers reported PHNs experiencing isolation and uncertainty when PHNs, during their role as school nurses, were trying to be ethically responsible and confront students who were misusing visual technologies and social media.^9^ Several studies had focused on how PHNs identify and resolve ethical challenges associated with technology in the learning environment^10^ and other issues in Norwegian school health services.^11^

Although few studies exist there are some qualitative investigations of the experiences of ethical conflict and moral distress of undergraduate students during their community nursing practice rotation. In the Philippines, students shared frustration over the disconnect between the ideals of nursing education such as equal access to health care and the practice reality, which contributed to their moral distress.^12^ US faculty and students also shared experiences of moral distress when unable to act ethically in the care of underserved and disadvantaged persons in a community-based education and practice program.^13^ Finally, there has been much news coverage of the enormous toll of the COVID-19 global pandemic on health professionals. Highlighted by ethics researchers have been findings of ethical anguish in community settings; for example, among PHNs in Korea who were responsible for pandemic prevention and management^14^ or, similarly, the moral distress of HCNs in Iran which manifested as feelings of helplessness and futility.^15^

## Research aim

The research aim was to identify the ethical conflicts experienced by community nurses (both HCNs and PHNs) who are employed in Canada.

## Research design

A qualitative research study^16^ was conducted by extracting interview data from a larger grounded theory project. The larger grounded theory project examined the process how community nurses manage ethical conflict and findings are published elsewhere.^1^ Participants had been asked during the larger project to first describe their ethical conflict experiences during their community nursing roles. By using qualitative content analysis (consistent with Graneheim and Lundman’s approach),^17^ we were able to identify from these extracted interview data the ethical conflicts experienced by community nurses.

## Research context and participants

Twenty-two participants were recruited through purposeful sampling^16^ from the province of Newfoundland and Labrador and two from the province of Ontario, Canada. Participants were selected because they were currently community nurses (either HCNs or PHNs) who had experienced one or more ethical conflicts during their practice. Other inclusion criteria were fluency in English, current professional registration/licensure as a registered nurse or nurse practitioner, and a minimum 1-year experience as a community nurse. Registered nurses employed in hospitals, Licensed Practical Nurses, and Personal Care Workers were excluded. Recruitment strategies included posting flyers in community health centers and public health units and asking managers to inform nursing staff about the research. Interested community nurses then contacted the research assistant (third author) directly. The sample of registered nurses (*n* = 24) comprised 11 HCNs and 13 PHNs. Twenty-three participants identified as female and one as male. Participants ranged in age from 23 to 57 years (mean age = 43 years) and had between 1.5 and 30 years (mean tenure = 30 years) experience in community nursing practice. Participants were given a small ($20) gift certificate of appreciation.

## Data collection

Three members of the research team conducted semi-structured interviews in-person in private office settings (*n* = 17), or if participants preferred, by telephone (*n* = 7). Interviews lasted approximately 60–90 min with the first part of the interview devoted to the community nurses describing their ethical conflicts. Questions included:

1. Can you describe a situation where you experienced ethical conflict due to a clinical situation? Tell me what happened.
2. How did that situation make you feel?
3. Whom did you talk to about this situation (if anyone)?
4. Did that clinical ethical conflict you described get resolved? If so, please describe the steps you took to resolve the issue. If this issue hasn’t yet been resolved, how are you managing this ethical conflict? What would you like to see happen? What makes the ethical conflict that you described, better or worse?

The interviews were audiotaped and then transcribed verbatim by the third author who was the research assistant and a professional transcriber who had signed a confidentiality agreement. To ensure participant confidentiality and security of the research data, participant identification codes and interview transcripts were kept separately in password-protected files on an encrypted computer in a locked office of the research assistant.

## Ethical considerations

We obtained research ethics approval from the Health Research Ethics Authority of Newfoundland and Labrador and from the health authorities where the community nurses were employed before initiating the project. We provided study information and clarified questions or concerns prior to receiving participant written consent. Anonymity and confidentiality were assured by removing all identifiers from the interviews during transcription and using numeric codes to identify participants. Anonymity, confidentiality, and the freedom to withdraw from the study at any time, were emphasized with participants.

## Data analysis

**Figure 1.**
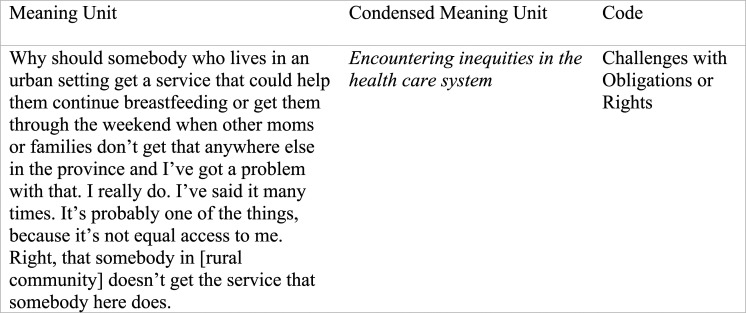
An example of the process of data analysis.

Whole interviews were the unit of analysis. Specifically, the broad descriptions of ethical conflicts shared by participants were the manifest content that we divided into content-related categories (what is referred to as the meaning units). We then interpreted what we thought were the types of ethical conflicts participants were describing and created the condensed meaning units. After applying Price’s^18^ typology of theories of ethics we were able to abstract the condensed meaning units and label with an overall code. See Figure 1 for an example of the process of analysis.

Strategies were used to ensure rigor—credibility, dependability, and transferability—as outlined by Graneheim and Lundman.^17^ For study credibility, we included community nurses working with a variety of populations in diverse settings and who represented a wide age range and career tenure. Nevertheless, as with virtually all research, the participants were community nurses who volunteered for the study and therefore we do not know if nurses who did not choose to volunteer for the study had different experiences. Study credibility was also promoted by ensuring that the community nurses had ample time to fully describe their experiences. In addition, the research team reviewed the verbatim transcripts to ensure data was not missed, took time to reflect on the clarity and meanings of the descriptions, and then met to discuss and agree on the final condensed meaning units and codes. Moreover, members of the research team upheld impartiality. That is, although they knew of colleagues and friends who had experienced ethical issues in community care settings, none of the team members were directly involved in community nursing practice, management, or policy, and as such had no undue influence on the study.

Verbatim transcripts included notations such as pauses or observed displays of emotion to promote understanding of the intended meaning. The analysis in terms of grouping and naming the ethical conflicts experienced by community nurses, was supported by providing ample quotations from participants. For dependability, semi-structured interview questions guided all participant interviews. To assist the reader with transferability of research findings, we identified the area of community nursing practice of each participant. In addition, the inclusion of multiple quotations in the findings not only serve to illustrate each ethical conflict but also enhance transferability.

## Findings

Normative and teleological ethics^18^ are philosophical approaches that guided identification of the types of everyday ethical situations extracted from interviews with community nurses. In total, seven ethical conflicts were identified. Four were labeled with the code “Challenges with Obligations or Rights” which aligns with normative ethics (focused on the standards of ethical behavior; e.g., the imperative obligations of nurses and the rights of patients). The three remaining ethical conflicts were labeled with the code “Challenges with Process, Risks, and Consequences” which aligns with teleological ethics (focused on what will a course of action mean; e.g., what would result if care was altered). The codes and their associated ethical conflicts are presented in Table 1.

**Table 1.** Presentation of final codes and associated ethical conflicts.

Codes	
Challenges with Obligations or Rights	Challenges with Process, Risks, and Consequences
*Ethical Conflicts*	*Ethical Conflicts*
1. *Screening for child developmental issues knowing there is a lack of timely early intervention services*	5*. Jeopardizing therapeutic relationships while reporting signs of risk*
2. *Encountering inequities in the health care system*	6*. Managing confidentiality when neighbors are clients*
3. *Not fulfilling principles, goals, and initiatives of primary and secondary prevention*	7. *Supporting client autonomy and decision-making but uncertain of the consequences*
4. *Feeling powerless to advocate for clients*	

### Challenges with obligations or rights

These ethical conflicts involved community nurses struggling with the right thing to do when wanting to fulfill professional obligations or struggling to provide care in accordance with client rights. A brief description is provided below of each ethical conflict identified.

#### Screening for child developmental issues knowing there is a lack of timely early intervention services

Several participants described experiencing an ethical conflict when making early intervention referrals because they knew that children would have to wait long periods of time. Despite complying with best practice guidelines that recommend early screening for child developmental issues and disabilities, participants described the reality of children not receiving developmental support and services; for example, appointments from speech therapists and speech-language pathologists were months and even years later after the initial referral. One of the participants (a PHN with 30 years experience) queried, “Is it ethical to refer if you know that, that child in the timeframe cannot be seen before they go to school?” Because of a lack of intervention services, child developmental issues were not being addressed in a timely manner. “That’s a huge ethical issue because it violates the principles of screening which are that you must have the services in place to deal with the issue for which you’ve identified. So really, all we’re doing right now is increasing parental anxiety and, also, we’ve got a group of children for whom they require services that we can’t deliver.” (PHN; 28 years experience)

#### Encountering inequities in the health care system

The community nurses described ethical conflicts that stemmed from an unfair distribution of health promotion programs and care supports and services. A PHN with 7 years experience claimed there was unequal access to services in the province and questioned, “Why should somebody who lives in an urban setting get a service that could help them continue breastfeeding or get them through the weekend when other moms or families don’t get that anywhere else in the province…?” Regions within the province were resourced differently. Residents in rural regions had fewer programs and services and had to travel long distances to an urban center for care that participants believed should have been provided. Cervical screening programs and diabetes education were in fact cut from a rural region of “very, very, very small communities” and are needs that remain unmet, as one of the participants explained.

I mean people still would go and get their pap smear somewhere potentially but in some cases it made it more inconvenient for them or they didn’t want to go to a male service provider instead of a female, or, you know, they had to travel longer distances. Um, and in terms of the diabetes, they, you know, in most cases with that, they did wait quite a bit longer because we were offering sessions every 2 weeks and because we could take smaller groups versus individuals at the hospital, they had to wait much longer. (PHN; 26 years experience)

#### Not fulfilling principles, goals, and initiatives of primary and secondary prevention

One of the participants complained that PHNs were not doing enough to address the social determinants of health by not acting, for example, on housing and food security issues at a program level. As a PHN with 28 years experience, she doubted whether the principles and goals of public health nursing were being fulfilled. While individual clients with housing issues could expect to have a PHN write letters to the housing corporation or speak to landlords and advocate on their behalf and defend their rights, there were no initiatives at a population level.

Moreover, while primary prevention initiatives to support families living in poverty such as the Healthy Beginnings Program are effective, the PHN is required to use a tool to assess families and assign scores. Several participants shared that the tool that includes financial status and income supports as criteria to gauge parenting capacity, compelled PHNs to prejudge parents: “Just the whole program, it’s like we decide who’s capable of raising their children. That’s the way I feel about it. And I feel like I don’t have any right to decide that….” (PHN; 5 years experience)

#### Feeling powerless to advocate for clients

Community nurses discussed several practice situations during which they wanted to advocate for clients’ perceived health and quality-of-life needs but felt powerless. Shared more than once was the obligation to arrange facility placement of older adults due to falls and injury, but because of the policies of long-term care and community support services, sometimes married couples had to be separated to accommodate different care need classifications (personal care homes are Levels 1 and 2; long-term care facilities are Level 3). It was ethically challenging to witness the separation of couples who had been together for decades, had never spent any time apart, and were deprived of the opportunity to even see each other unless a family member was able to organize a visit.

Also ethically challenging was the obligation to arrange for the transfer of clients who are older adults without dementia to long-term care facilities or personal care homes within which clients would be cohabitating with residents who have dementia or some form of cognitive impairment. There are reports of residents who after transitioning to their new living environments were feeling anxious and fearful because of the erratic behaviors of other residents brought on by cognitive degeneration and the resultant mental instability. The community nurse shared, “I have another resident in another home who had a brain injury, flipped his quad, had a brain injury. He’s creating havoc! He’s in a personal care home with a bunch of seniors and he’s inappropriate and he’s cursing and swearing and he’s belligerent.”

Feeling powerless to advocate for end-of-life clients in the home setting was another example of an ethical conflict for community nurses. Participants discussed their inability to advocate on behalf of clients wishing to die at home when a physician insists that the client be transported to the hospital; or when there are insufficient in-home palliative care services to support family caregivers; or during cases when family members or the physician are reluctant to administer narcotic pain relief medications. Subsequently, clients suffered: “I did have a situation probably recently within this position. I had a physician that was reluctant to order pain meds for a palliative client or increase the pain medication. I felt the client was suffering because he was in pain.” (HCN and PHN; 18 years experience)

### Challenges with process, risks, and consequences

Challenges with process, risks, and consequences refer to ethical conflicts during which community nurses deliberate over what may happen as a result of certain care measures. A brief description is provided below of each ethical conflict identified.

#### Jeopardizing therapeutic relationships while reporting signs of a child at risk

Establishing therapeutic relationships with parents is critical to ensuring PHNs can access and assess home environments of children at risk. When signs of neglect or abuse arose, participants were troubled knowing that reporting families to Child Protection and Youth Services (CPYS) would jeopardize future rapport with parents.

… sometimes people do tell you things in confidence sometimes and then they don’t realize that this is something that really needs to be brought to the attention of CPYS and you know I’ve actually had people say that to me. That like, ‘You know I trusted you with this information. Well, you know I understand that but you know if it comes down to affecting the care of a child then I do have to report that’ and then unfortunately what happens then is that your relationship ends pretty much with that client because they don’t really want anything else to do with you. (PHN; 19 years experience)

One of the participants with 7 years experience stated that building the relationship takes considerable time and then having “to lose that relationship” and betray trust to fulfill the duty to report after “doing well with them,” is “probably one of the hardest things” in her role as a PHN.

#### Managing confidentiality when neighbors are clients

Navigating the challenges of maintaining confidentiality when living and working in a small community emerged several times during the interviews. Participants, for example, stated managing confidentiality meant avoiding conversations with friends and family members in the grocery store or with neighbors seeking advice, and rejecting Facebook messages containing inquiries about health issues. One of the PHNs with 25 years experience shared how she would manage a typical encounter in a public space.

Okay, so what do you do with that in the middle of the mall when two people approach you and say, ‘Oh hi you’re the nurse that visits my sister. We think she’s got postpartum depression and needs help.’ And you know what the consequences of postpartum depression can be in terms of the baby and things like that. So, they said, ‘Can you call her?’ And I said, ‘Well no, I can’t because you know, she needs to call me or you need to tell her that you’ve talked to me.’

Addictions counseling and follow-up reporting and partner notification for sexually-transmitted infections (STIs) were especially challenging, as disclosed by a PHN.

… and then like STI follow-ups are hard too because like I might not know a person well but I know most of the referrals that I get. So, then you’re calling them, they know you know them and they know you know that they have a STI, so. But it’s like you can’t give away all the ones that you know, know what I mean. I can’t give them all to the other PHNs because they are from here too. So those times you just got to be so, and I stress to them like that, ‘It is all confidential,’ and you know and, ‘I will never ever say anything.’

#### Supporting client autonomy and decision-making but uncertain of the consequences

Participants who were responsible for childhood immunization programs discussed the difficulty supporting parental autonomy and decision-making when parents refused to vaccinate their children. A PHN with 19 years experience explained,

… another big ethical conflict for probably all PHNs is when parents refuse immunizations. …we have to respect people’s, you know, ability to make their own decisions but sometimes that, you know, it creates an internal conflict because you know that child is at risk for communicable diseases and they are also putting other people at risk….

Some PHNs interviewed believed that parents are reading “tainted” and invalid website information and relying on misinformation on Facebook. One of the PHNs with 26 years experience disclosed how she thought she was resorting to “scaremongering” tactics when she exposed parents to articles (from Centers for Disease Control and Prevention) of children with tetanus and other diseases because of their parents’ antivaccination stance. While taking this action to encourage parents to change their decision she questioned if she was going too far, “pushing them too strongly,” and “trying to influence their decision.”

Similarly, the HCNs expressed difficulty supporting the autonomy and decision-making of clients wishing to live independently in their home without what HCNs deemed were needed services.

… alot of the focus on community health is autonomy, to let people make their own decisions. I felt like when I was in nursing school when we were in acute care it was kind of go out and fix them and do whatever you can to fix the situation. When you’re in community you have to kind of let patients take responsibility for themselves as much as they’re able. (HCN; 11 years experience)

HCNs wanted to foster self-determination and they acknowledged that in-home care support and services are voluntary. However, in some cases there was no certainty that clients would and could make the safest choices as depicted in a situation shared by one of the HCNs.

So, we just started visiting her more often and you know the more you would see her I guess, the more you’d, you know, the more issues we were coming up with. And then there was always this, I guess this would be your ethical dilemma, of you know, she was a very proud lady. She seemed like she was coping. She was bothered sometimes by the fact that we were bothering her is what she would look at it as.

## Discussion

It is evident from our research that lack of resources due to economic constraints in the provision of community-based health care, is a major factor undermining the community nurses’ abilities to fulfill nursing obligations or to protect client rights. It is under these circumstances that ethical conflicts arose such as PHNs *screening for child developmental issues knowing there is a lack of timely early intervention services.* Early intervention is a systematic approach for identifying and coordinating child developmental supports during the crucial first 5 years and may include applied behavioral analysis, speech-language therapy, and occupational therapy, among others. It is not uncommon for parents to wait 2 years for a speech-language therapist. Long wait times, a defining characteristic of the Canadian health care system,^19^ lead some parents to pay out of pocket for services. A recent news report indicated that parents were required to take second mortgages on their homes to cover costs of private services with annual fees ranging from $25,000 to $80,000.^20^ Parents who could afford to pay privately had the advantage and did not have to wait which was an obvious disparity that confronted the community nurses we interviewed and that is also reported elsewhere.^21^

Economic constraints were also responsible for the widespread disparity in terms of equitable access to care across the province of Newfoundland and Labrador. *Encountering inequities in the health care system* is another ethical conflict that was encountered by several community nurses. According to the Canada Health Act’s^22^ principles of accessibility and comprehensiveness, the complete range of publicly-insured health services should be available to all citizens. Although the principles underpin their ethical practice, community nurses constantly struggled with the existing discrepancy between what they could offer to residents in an urban region in contrast to what was accessible and available in rural communities. Davidson, Rushton, Kurtz, et al.^23^ recommend a social-ecological framework to make sense of ethical conflicts and to inform ethical practice barriers by illuminating the nurse’s ecosystem, including social, cultural, political, and economic contexts. Ethical dilemmas are created when nurses must account for economic and other systemic constraints^18^ so that arming community nurses with understanding of the interplay of competing factors in an organizing framework may contribute to formulating interventions for real change. Taking action for real change to address the social determinants of health inequalities, including socioeconomic status, is something the PHNs aspired as they discussed their ethical conflict—*not fulfilling principles, goals, and initiatives of primary and secondary prevention*.

According to the Census of Population survey by Statistics Canada, one in five Canadians was 65 years or older in 2021^24^ and this aging demographic is expected to accelerate and with it the increasing demand for community-based health care services. Older adults were at the center of examples of ethical situations cited by HCNs when HCNs expressed *feeling powerless to advocate for clients* to ensure the best quality of life. Barriers were associated with finite resources and departmental policies that were beyond the purview and control of HCNs and better handled at managerial levels. Managers are critical to successful resolution of ethical dilemmas and can play a key role in mitigating ensuing anxiety and guilt and other symptoms of moral distress that HCNs may carry if devoid of opportunity for debriefing and deliberation.^1^ Interestingly, Norwegian researchers^8^ well aware of the everyday ethics of community care, discovered that managers who were overseeing long-term care staff and HCNs perceived that ethical conflicts were an individual and personal staff responsibility and failed to recognize the significance of ethical leadership and organizational factors in supporting ethical competence.

Also made evident from our research is the staunch defense by community nurses of nursing values and ethical responsibilities. *Supporting client autonomy and decision-making but uncertain of the consequences* emerged as an ethical conflict facing PHNs and HCNs because while unwilling to compromise the Code of Ethics’ “promoting and respecting informed decision-making,”^2^ they were unsure in some cases if client decisions were in the best interests of clients or the community. Ethical issues such as these will arise. Given the complexity of community-based health care the Code of Ethics cannot be applied in a “static way” because there are “multiple goods” (e.g., respecting the parental decision not to vaccinate a child and being mandated to prevent childhood disease) that need to “co-exist.”^10^ In cases of suspected child abuse or neglect, however, community nurses told us that they could be resolute in their decision to take action and prioritize the health and well-being of the child but then they inevitably faced the ethical conflict of *jeopardizing therapeutic relationships while reporting signs of a child at risk.* Finally, *managing confidentiality when neighbors are clients* is the seventh and last ethical conflict that we identified and pertains to those community nurses who shared that they are small-town residents which meant that their personal and professional lives often collided. However, they developed several strategies and became adept at “maintaining privacy and confidentiality”^2^ when clients were neighbors, friends, or family members.

## Strengths and limitations

Recruitment of both HCNs and PHNs captured a wide range of ethical conflicts that may resonate with community nurses in jurisdictions across Canada as well as beyond international boundaries. Some of the community nurses who participated told the team that they did so because they noted that most studies about nurses’ work life examined hospital nurses and they wanted more to be known about the everyday realities and complexities of community nursing practice. However, as mentioned previously, participants were those community nurses who willingly stepped forward. There may have been some community nurses who for various reasons were hesitant to share their ethical issues and one wonders if those ethical challenges were the same or different.

There was only one male participating in the study which is not unexpected given that approximately 90% of Canadian nurses are female.^25^ Further, the percentage of nurses who are male in the province of Newfoundland and Labrador where most of the interviews were conducted, is lower than the Canadian average. Moreover, male nurses are more apt to work in acute care institutions; for example, in intensive care units and in emergency departments as opposed to specialties in community settings.^26^

## Conclusions

It is evident from our research findings that community nurses in Canada are experiencing ethical conflicts and while there are similarities, the conflicts are significantly different from the ethical situations and dilemmas encountered by acute care nurses. It is also apparent that inequitable allocation of limited resources is one of the root causes and a major contributing factor to the ethical conflicts experienced by community nurses. Limited resources are the barriers, for example, to fostering healthy child development, to community health promotion programming, and to mitigating the effects of the social determinants of health. During support and care in the home setting community nurses must resolve ethical conflicts that arise from other social and political factors in the organizational health care context that challenge their ability to uphold client rights, such as client autonomy. Each and every day community nurses will continue to experience challenges to their ethical practice especially as demand for community-based health care increases due to Canada’s aging demographics, high morbidity rates, and amid the mental and physical stress of economic recession.

Affecting Canada and countries around the world is the real threat of emerging viruses as we witnessed during the global pandemic. Although our research was conducted prior to the coronavirus (COVID-19) outbreak, we know from news outlets that community nurses were profoundly impacted during the pandemic by deficiencies of both material and human resources and by other inhibiting factors beyond their control. Future research on the topic of ethical conflicts facing community nurses will be critical to raise awareness and understanding both in Canada and beyond to advance ethical competence.
